# Apoptin Regulates Apoptosis and Autophagy by Modulating Reactive Oxygen Species (ROS) Levels in Human Liver Cancer Cells

**DOI:** 10.3389/fonc.2020.01026

**Published:** 2020-06-25

**Authors:** Yiquan Li, Yilong Zhu, Jinbo Fang, Wenjie Li, Shanzhi Li, Xing Liu, Zirui Liu, Gaojie Song, Chao Shang, Jianan Cong, Bing Bai, Lili Sun, Ningyi Jin, Xiao Li

**Affiliations:** ^1^Academician Workstation of Jilin Province, Changchun University of Chinese Medicine, Changchun, China; ^2^Institute of Military Veterinary Medicine, Academy of Military Medical Science, Changchun, China; ^3^Department of Head and Neck Surgery, Tumor Hospital of Jilin Province, Changchun, China; ^4^Jiangsu Co-innovation Center for Prevention and Control of Important Animal Infectious Diseases and Zoonoses, Yangzhou, China

**Keywords:** human liver cancer, apoptin, apoptosis, autophagy, ROS

## Abstract

Apoptin is a protein that specifically induces apoptosis in tumor cells. The anti-tumorigenic functions of Apoptin, including autophagy activation and its interaction with apoptosis, have not been precisely elucidated. Here we investigate the main pathways of apoptin-mediated killing of human liver cancer cells, as well as its putative role in autophagy and apoptosis. The anti-proliferative effect of apoptin in liver cancer cells was analyzed *in vitro* by crystal violet staining and MTS detection, and also *in vivo* using a tumor-based model. The main pathway related to apoptin-induced growth inhibition *in vitro* was evaluated by flow cytometry and fluorescence staining. The relationship between apoptosis and autophagy on apoptin-treating cells was analyzed using apoptosis and autophagy inhibitors, mitochondrial staining, Annexin V-FITC/PI flow detection, LC3 staining, and western blotting. The effect of ROS toward the apoptosis and autophagy of apoptin-treating cells was also evaluated by ROS detection, Annexin V-FITC/PI flow detection, LC3 staining, and western blotting. Inhibition of apoptosis in apoptin-treating liver cancer cells significantly reduced the autophagy levels *in vitro*. The overall inhibition increased from 12 h and the effect was most obvious at 48 h. Inhibition of autophagy could increase apoptin-induced apoptosis of cells in a time-dependent manner, reaching its peak at 24 h. Apoptin significantly alters ROS levels in liver cancer cells, and this effect is directly related to apoptosis and autophagy. ROS appears to be the key factor linking apoptin-induced autophagy and apoptosis through the mitochondria in liver cancer cells. Therefore, evaluating the interaction between apoptin-induced apoptosis and autophagy is a promising step for the development of alternate tumor therapies.

## Introduction

Liver cancer is the second leading cause of cancer-related death in the world. In January 2018, the American Cancer Society reported that the total cancer death rate had slowly dropped by 25% for the past 25 years in USA, but the mortality rate due to liver cancer was still rising (1). It has been estimated that ~700,000 new liver cancer patients are diagnosed and 600,000 patients die due to this disease every year in the world (1). Only in China, more than 300,000 people die of liver cancer every year, which accounts for about half of the global liver cancer deaths (2). Currently, surgical resection is still one of the most effective methods for treatment of the disease. However, its long-term efficacy is still poor. Therefore, finding effective and poorly toxic drugs would be the choice for treating liver cancer.

The specific induction of programmed cell death (PCD) in tumor cells has great potential for treating tumors. Great efforts have been made in the study of tumor suppressor genes and proteins, such as apoptin, TRAIL, and MDA-7, which seem to specifically kill tumor cells (3, 4). The chicken anemia virus (CAV) VP3 protein is a 13.6 kDa serine-threonine-rich protein containing 121 amino acids with the ability to induce PCD in chicken thymocytes (5). Since the CAV VP3 protein also has the ability to induce cell death and apoptosis, it has been dubbed “apoptin” (6, 7). Apoptin is a protein that can specifically induce tumor cell apoptosis, it does not lead to programmed cell death in most of the normal cells (8, 9). Since this protein is neither regulated by p53 nor inhibited by Bcl-2 overexpression, apoptin has been considered a novel anti-tumor biological protein (10–12).

Apoptosis is typically mediated by (i) an intracellular caspase, (ii) a “promoter,” and (iii) an “executor” of the apoptotic process (13, 14). Caspase activation is accomplished by two major signaling pathways: the extrinsic (or death receptor) pathway and the intrinsic (or mitochondrial) pathways. In the extrinsic pathway, the binding of homologous ligands to death receptors (DRs) triggers the recruitment of the adaptor protein FADD and the apoptotic promoter caspase-8 to the death-inducing signaling complex (15). The intrinsic pathway is initiated by the release of cytochrome c, a process closely regulated by pro- and anti-apoptotic members of the Bcl-2 family (16). Upon release, cytochrome c binds to the adaptor protein Apaf-1, allowing the initiator caspase-9 to be activated in cell fragments called apoptotic bodies (17). These two pathways converge to the activation of downstream effector caspases, leading to cell death by cleavage of several cellular substrates (18).

Apoptin-mediated cell death is not associated with DRs, since cells lacking FADD or caspase-8 are still sensitive to apoptin (19). Furthermore, blocking the death receptor CD95 with a neutralizing antibody does not affect apoptin-induced cell death (19). Unlike the composition of the extrinsic pathway, apoptin-mediated apoptosis is strongly influenced by regulators of the intrinsic pathway. For instance, Apaf-1 deficiency has a strong protective effect in tumor cells (20).

Autophagy is a “cell waste treatment” process in which cells use lysosomes to degrade their damaged organelles and macromolecules, under the regulation of autophagy-related genes (Atg) (21). Studies have shown that autophagy largely occurs when cells are under starvation, hypoxia, endoplasmic reticulum stress, and radiation (22–25). Autophagy is very important for maintaining homeostasis, however, excessive cell damage can activates autophagy and turn it into a form of cell death (also called type II programmed cell death) (26). So, both autophagy and apoptosis can together regulate a broad range of cell death events. Still, in the context of apoptin-induced, cell death, autophagy activation, and an interaction with apoptosis have not been precisely elucidated.

In our previous study, a recombinant type 5 adenoviral vector overexpressing Apoptin (Ad-apoptin) was constructed. This delivery system has been shown to efficiently (and consistently) express apoptin protein in cells (27). In fact, ectopic expression based on the use of transfection reagents have a few disadvantages, including (i) lack of experimental consistency and (ii) expression decline due to cytotoxic effects and/or low efficiency and instability of the reagent. Here we utilize the same adenoviral system to analyze and study major pathways of apoptin-mediated cytotoxicity in human liver cancer. Specifically, we have evaluated the role of apoptin in the activation of autophagy as well as its interaction with apoptosis, to strengthen the theoretical basis of apoptin and its potential use in cancer therapy.

## Materials and Methods

### Cells, Viruses, and Animals

Human liver cancer cells SMMC-7721 were acquired from the Cell Bank of the Shanghai Institute for Biological Sciences (Shanghai, China). The recombinant adenoviruses Ad-apoptin and Ad-mock were constructed and preserved in our laboratory (Laboratory of Molecular Virology and Immunology, Institute of Military Veterinary Medicine, Academy of Military Medical Science, Changchun, China) (27).

Female BALB/c nude mice aged 4–5 weeks were purchased from the Experimental Animal Center of the Academy of Military Medical Sciences of China. All animal experimental protocols were approved by the Institutional Animal Care and Use Committee (IACUC) of the Chinese Academy of Military Medical Science (Changchun, China) (10ZDGG007). All surgeries were performed under anesthesia with sodium pentobarbital, according to standard animal procedures.

### Crystal Violet Staining

Liver cancer cells were infected with recombinant adenoviruses (Ad-apoptin or Ad-mock) at a dose of 100 MOI. Cell cultures were processed at 12, 24, and 48 h post-infection, respectively. For this, culture medium was discarder out and cells were washed three times with PBS, followed by staining with 0.4% crystal violet (500 μl of dye solution per well) for 5 min at room temperature. Thereafter, dye solution was removed, stained cells were washed three times with PBS, and plates were then dried at room temperature before analysis.

### MTS Assay

Liver cancer cells were infected with recombinant adenoviruses (Ad-apoptin or Ad-mock) at a dose of 10 or 100 MOI. Cell cultures were then processed at 6, 12, 24, 48, and 72 h post-infection, respectively. MTS reagent was added at 20 ug/well, and then cells were cultured accordingly for further 2 h. Respective OD values were measured at 490 nm using a microplate reader. Inhibition of cell proliferation/viability (cell suppression rate) was calculated as the following: Cell suppression rate = 100% × (1 - absorbance of treated wells/absorbance of control wells).

### Tumor Growth *in vivo* (Xenograft)

SPF female BALB/c nude mice were fed with SPF-grade sterilized rat diet and water in a sterile environment. Animals were subjected to adaptive feeding for 7–10 days.

Mice were injected subcutaneously with 100 μl (5 × 10^7^ cells/mL) of Liver cancer cells in the right hind limb (near the back area). After successful tumor-bearing, nude mice were randomly divided into three groups, namely Ad-apoptin, Ad-mock, and control groups. Tumor size was measured once a week (up to 6 weeks) using a vernier caliper. Tumor volume was calculated as the following: Tumor volume = a^2^ × b × 0.5 (a = short diameter of the tumor; b = tumor length). An average tumor growth curve was then plotted. According to each group, purified recombinant adenoviruses were injected into the tumor mass every 3 days for six times (5 × 10^8^ PFU/100 μl/intratumor injection). The relative inhibition rate of tumor growth was calculated, and an average tumor inhibition curve was plotted. Survival was recorded every day for 6 weeks. A graph indicating survival time (in days) vs. survival rate was further plotted.

### Hoechst Staining Assay

Liver cancer cells were infected with recombinant adenoviruses (Ad-apoptin or Ad-mock) at a dose of 100 MOI. In addition, 20 μM QVD (apoptosis inhibitor) was administered to an Ad-apoptin group. Cell cultures were processed at 12, 24, and 48 h post-infection, respectively. Thereafter, culture solution was discarded and plated cells were washed three times with PBS. Cells were then digested with 0.25% trypsin, and both digested cells and original culture solution were centrifuged at 500 × g for 5 min. After discarding the supernatant, cell pellet was washed three times with PBS, followed by the addition of 1 ml Hoechst dye solution at 10 μg/ml. Cells were stained for 15 min in the dark, then centrifuged at 500 × g for 5 min and washed as previously. A total of 100 μL of DMEM was used to resuspend the cell pellet. About 10 μl of the stained cell mixture was transferred to a glass slide, gently covered with a coverslip and then analyzed by fluorescence microscopy.

### Annexin V-FITC/PI Flow Detection

Liver cancer cells were infected with recombinant adenoviruses (Ad-apoptin or Ad-mock) at a dose of 100 MOI. Ad-apoptin groups were treated with 20 μM QVD, autophagy inhibitor 3-MA (5 mM 3-Methyladenine), CQ (20 μM Chloroquine), or ROS inhibitor (10 mM NAC), respectively. Cell cultures were processed at 6, 12, 24, and 48 h post-infection, respectively. Thereafter, culture solution was discarded and plated cells were washed three times with PBS. Cells were then digested with 0.25% trypsin, and both digested cells, and original culture solution were centrifuged at 500 × g for 5 min. After discarding the supernatant, cell pellet was washed three times with PBS, followed by the addition of 5 μl FITC and 5 μl PI. Samples were stained in the dark for 20 min, at room temperature. Samples were then transferred to the flow tube and properly labeled before flow cytometry.

### Detection of Mitochondrial Membrane Potential

Liver cancer cells were infected with recombinant adenoviruses (Ad-apoptin or Ad-mock) at a dose of 100 MOI. At the same time, the apoptosis inhibitor QVD (20 μM) was administered to an Ad-apoptin group. Cell cultures were processed at 12, 24, and 48 h post-infection, respectively. Thereafter, culture solution was discarded and plated cells were washed three times with PBS, followed by staining with JC-1 dye solution at 1 mM. Cells were stained for 15 min in the dark and then washed three times with PBS. Cell slides were further mounted and analyzed by fluorescence microscopy.

For quantitative measurement, cells were plated into 96-well-plates, and then cultured as previously. Experimental procedures followed as indicated for the qualitative analysis. Plated cells were washed three times with PBS, followed by staining with 100 μl of JC-1 dye (1 mM solution) for 15 min. OD values were measured at 485–530 and 530–590 nm using a microplate reader.

### Western Blotting

The liver cancer cells were infected with recombinant adenoviruses (Ad-apoptin or Ad-mock) at a dose of 100 MOI. At 6, 12, 24, and 48 h post-infection, cells were trypsinized, and collected by centrifugation at 5,000 rpm for 5 min. Cell pellets were resuspended in lysis buffer and protein solution was collected by centrifugation at 12,000 rpm for 5 min. All samples were analyzed by western blotting (28).

### LC3 Immunofluorescence Assay

The liver cancer cells were infected with recombinant adenoviruses (Ad-apoptin or Ad-mock) at a dose of 100 MOI. At 6, 12, 24, and 48 h post-infection, the cells were fixed with 4% paraformaldehyde in PBS. The cells were blocked in 5% non-fat dried milk in PBS supplemented with 0.1% tween 20 (PBST) and then incubated overnight with the corresponding primary antibody at 4°C overnight. The blots were then incubated with respective secondary antibody for 1 h after three washes with PBST. Cellular images were then obtained using a fluorescence microscope.

### pEGFP-LC3 Plasmid Transfection

The liver cancer cells were transfected with pEGFP-LC3 using the X-treme GENE HP DNA Transfection Reagent for 24 h. After the designated treatments, the cells were fixed with 4% paraformaldehyde in PBS. Cellular images were then obtained using a fluorescence microscope.

### Lysosomal Staining Assay

Liver cancer cells were infected with recombinant adenoviruses (Ad-apoptin or Ad-mock) at a dose of 100 MOI. Ad-apoptin groups were treated with QVD or NAC (n-acetyl cysteine), respectively. Cell cultures were processed, as previously, at 6, 12, 24, and 48 h post-infection, respectively. Cell suspensions were centrifuged at 500 × g for 5 min, and resulting cell pellets were resuspended with 1 ml of 100 nM LTR (Lyso-Tracker Red) dye solution. Staining was performed for 30 min in the dark. Stained cells were centrifuged at 500 × g for 5 min, followed by three washes with PBS. Final pellets were resuspended in 100 μL DMEM. A 10 μl aliquot of labeled cell mixture was transferred to a glass slide, gently covered with a coverslip, and finally observed by fluorescence microscopy.

### TMRM Staining Assay

Liver cancer cells were infected with recombinant adenoviruses (Ad-apoptin or Ad-mock) at a dose of 100 MOI. Ad-apoptin cells were treated with 3-MA and CQ (autophagy inhibitor). Cell cultures were processed at 6, 12, 24, and 48 h post-infection, respectively. The cells were washed three times with PBS, followed by staining with TMRM dye (10 μg/ml solution) for 15 min in the dark. Stained cells were washed three times with PBS and centrifuged at 500 × g for 5 min. Cell pellets were further resuspended with 1 ml of Hoechst dye (10 μg/ml solution) and re-incubated for 15 min in the dark. Cells were then washed three times with PBS. Cell slides were further mounted and analyzed by fluorescence microscopy. After the above treatment, samples could also be transferred to pre-labeled flow tubes for flow cytometry.

### Observation on Colocalization of Mitochondria and LC3

The liver cancer cells were infected with recombinant adenoviruses (Ad-apoptin or Ad-mock) at a dose of 100 MOI. At 12 and 48 h post-infection, the cells were washed three times with PBS, followed by staining with MTG (Mito-Tracker Green) dye (100 nM solution) for 15 min in the dark and then cells were fixed with 4% paraformaldehyde in PBS. The cells were blocked in 5% non-fat dried milk in PBS supplemented with 0.1% tween 20 (PBST) and then incubated overnight with the corresponding primary antibody at 4°C overnight. The blots were then incubated with respective secondary antibody for 1 h after three washes with PBST. Cellular images were then obtained using a fluorescence microscope.

### Observation on Colocalization of Mitochondria and LC3/Lysosomes

Liver cancer cells were infected with recombinant adenoviruses (Ad-apoptin or Ad-mock) at a dose of 100 MOI. Ad-apoptin cells were treated with 3-MA and CQ (autophagy inhibitor). Cell cultures were processed at 12 and 48 h post-infection, respectively. The cells were washed three times with PBS, followed by staining with MTG (Mito-Tracker Green) dye (100 nM solution) for 15 min in the dark. Stained cells were washed three times and stained with 1 ml of LTR dye (100 nM solution) and re-incubated for 15 min in the dark. Cells were then washed three times with PBS. Cell slides were further mounted and analyzed by fluorescence microscopy.

### ROS Detection

Liver cancer cells were infected with recombinant adenoviruses (Ad-apoptin or Ad-mock) at a dose of 100 MOI. Ad-apoptin groups were treated with QVD (apoptosis inhibitor), 3-MA, and CQ (autophagy inhibitor), respectively. Cell cultures were processed, as previously, at 6, 12, 24, and 48 h post-infection. Cell were resuspended as described, and further centrifuged at 500 × g for 5 min. The cell pellets were washed three times with PBS, then resuspended with 1 ml of DHR dye (1 mM solution) and incubated for 30 min in the dark. Stained cells were centrifuged and then washed as before. Cell pellets were resuspended in 500 μL fluorescent fixative before analysis by the flow cytometry.

### Statistical Analysis

Statistical analysis was conducted using data from at least three independent experiments, with SPSS 20.0 (SPSS Inc., Chicago, IL, USA). Results were statistically analyzed by Student's *t*-test or one-way analysis of variance (ANOVA; *p* < 0.05). Multiple comparisons were performed using Student-Newman-Keuls test. A *p* < 0.05 was used as a threshold for statistical significance.

## Results

### Inhibitory Growth Effect of Apoptin in Liver Cancer Cells

**Figure 1:**
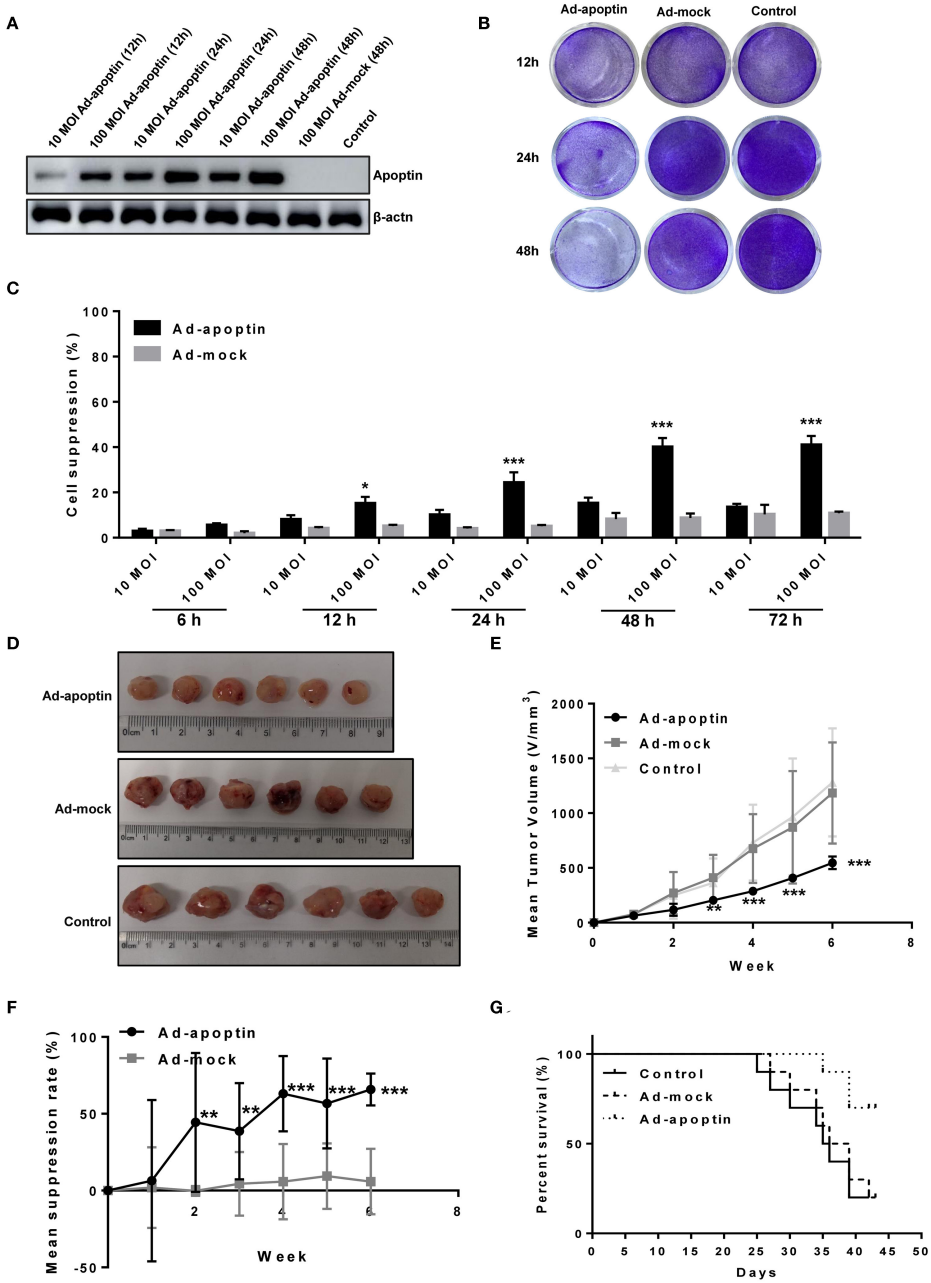
Inhibitory effect of apoptin on SMMC-7721 cells *in vitro* and *in vivo*. **(A)** Western blotting analysis of apoptin protein expression at different time points and different virus titers. 100 MOI of Ad-apoptin expresses apoptin protein more effectively than 10 MOI, and with the increase of time, the expression level also increases; the Ad-MOCK and control group do not express apoptin. **(B)** SMMC-7721 cells were infected with Ad-apoptin or Ad-mock at 100 MOI, then stained with 0.4% crystal violet at 12, 24, and 48 h. Ad-apoptin exerts inhibitory effects on SMMC-7721 cells. **(C)** Viability of SMMC-7721 cells was determined using MTS assays, after infection with Ad-apoptin or Ad-MOCK at two different doses (10 and 100 MOI) for 6, 12, 24, 48, and 72 h. The inhibitory effect of Ad-apoptin had a significantly higher than other groups on liver cancer cells. All measurements were performed in triplicates. **(D,E)** Xenograft models were established via subcutaneous injection of SMMC-7721 cells (5 × 10^6^/100 μL) into the right hind limb of the mice (*n* = 10 per group). Length and width of xenograft tumors measured weekly, for 6 weeks, using vernier caliper. The tumor growth rate of Ad-apoptin group was significantly lower than that of other groups. **(F)** Average tumor inhibition was calculated using the formula: (1 - treatment group tumor weight/control tumor weight) × 100%. In the last week, the average tumor inhibition of the Ad-apoptin group was about 62.32%. **(G)** After successfully establishing xenograft models in nude mice, the survival of mice was recorded every day for 6 weeks. The survival rate of the Ad-apoptin group was also the highest, reaching 70.00%. Data are presented as means ± SD (^*^p < 0.05, ^**^p < 0.01, ^***^p < 0.001) compared with Ad-mock or controls.

We tested the expression of apoptin protein of Ad-apoptin at different time points and different doses (Figure 1A). The result showed that the apoptin level was changed with the time and does, and the longer the time and does, the more expression. Subsequently, crystal violet staining was performed to allow a more qualitative analysis of cell viability (Figure 1B). For this, a recombinant apoptin-expressing adenovirus (Ad-apoptin) was inoculated into SMMC-7721 cells and, at 12, 24, and 48 h post-infection, cells were subjected to crystal violet staining. These results indicate that Ad-apoptin can lead to a time-dependent inhibitory effect after infection, while a control adenovirus (Ad-mock) showed no significant effect. Therefore, the expression of apoptin can promote the killing effect on liver cancer cells to a certain extent.

The cell suppression rate of adenovirus infected liver cancer cells was further quantified by MTS assay at different times after infection (Figure 1C). Ad-apoptin induced distinct inhibitory effects in SMMC-7721 cells, while Ad-mock showed no inhibitory effect in cells. At 12 h post-infection, the growth rate inhibition induced by Ad-apoptin in liver cancer cells significantly increased, and this effect was significantly higher than the Ad-mock group (*P* < 0.05). Gradually, the inhibitory effect of Ad-apoptin *in vitro* increased, reaching the highest level after 48 h of infection. At 72 h post-infection, the cell suppression rate of Ad-apoptin was maintained at a similar levels to that at 48 h (40.25%). The inhibitory effect of Ad-apoptin at MOI of 100 was significantly higher than at MOI of 10. These results suggest that Ad-apoptin has a significant inhibitory effect in the growth of human liver cancer cells and this inhibition is dose- and time-dependent.

### *In vivo* Inhibitory Effect of Apoptin on Liver Cancer Cells

The results of the tumor diameter measurement after subcutaneous injection of cancer cells *in vivo* are shown in Figures 1D–F. The size of tumor xenografts in the Ad-apoptin, Ad-mock, and control mice groups showed a trend of gradual increase after tumor-bearing stage. The sizes of Ad-mock and control tumor xenografts were similar and developed at fast growth rate. Contrarily, the size of tumor xenografts in the Ad-apoptin group started to decrease in the first week after tumor-bearing. As a result, the size of tumor xenografts in the Ad-apoptin group was significantly smaller than those in the control group after second week of implantation. Until the end of the experiment, the tumor growth in the Ad-apoptin group was still very slow. In the last week, the average inhibition of the Ad-apoptin group was about 62.32%. These observations reiterate the notion that apoptin has a potential antitumor activity *in vivo*.

The survival of mice was observed and recorded since tumor-bearing (see results in Figure 1G). The Ad-mock and control groups deceased by the 25th and 27th day after tumor-bearing. The average survival time of Ad-mock and control groups was about 32.4 and 33.5 days after injection of SMMC-7721 cells. The difference between these results was not significant (*P* > 0.05). The survival of mice from the Ad-apoptin-treated group was prolonged, with an average survival time of ~42.5 days after injection of SMMC-7721 cells. This result was significantly different from the Ad-mock-treated and the control groups (*P* < 0.05). The survival rates in the Ad-apoptin-treated group were 70%. This indicates that apoptin can significantly improve survival rate after implantation of liver cancer cells.

### Apoptin Induces Apoptosis of Liver Cancer Cells

**Figure 2:**
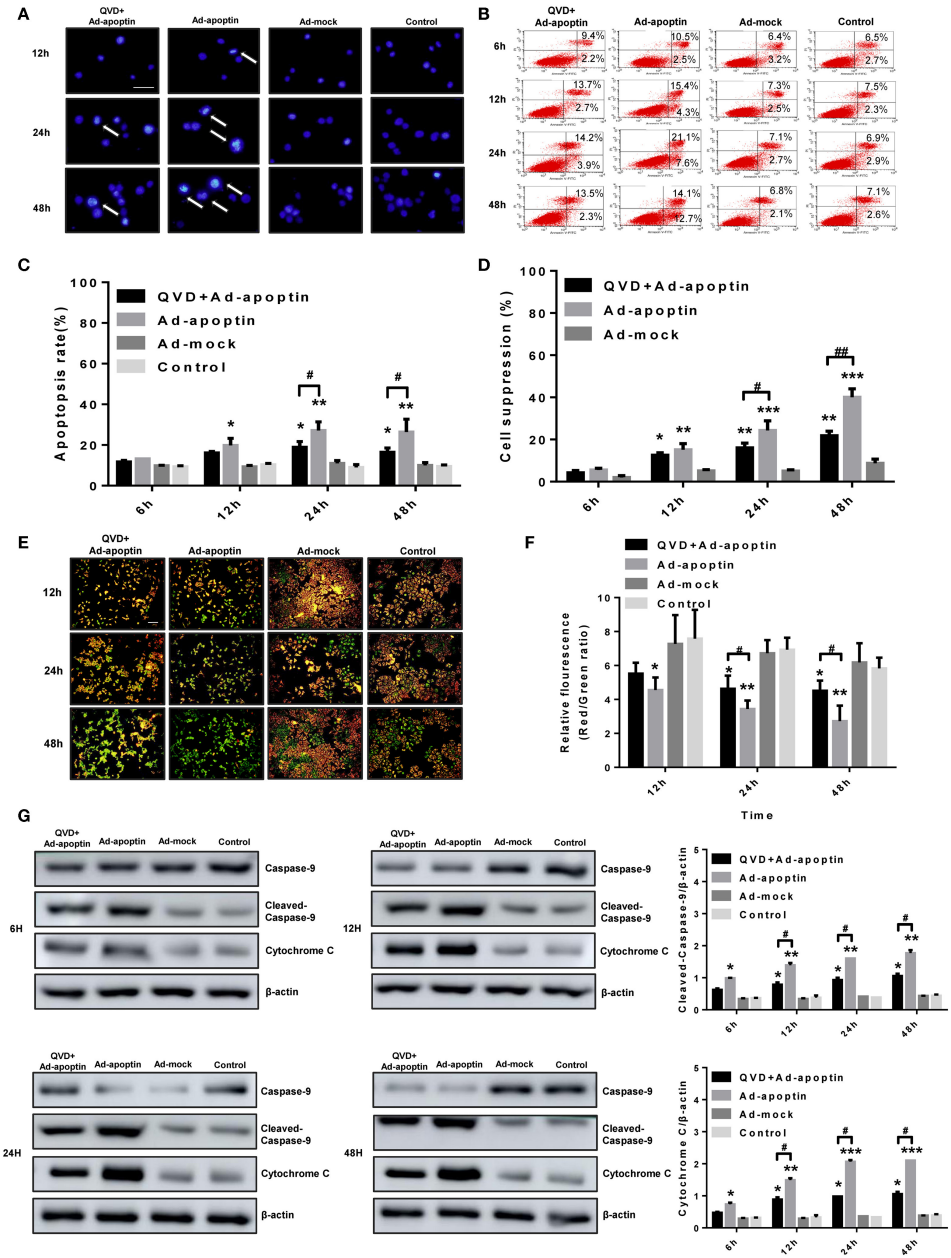
Characterization of cell death pathway induced by apoptin in SMMC-7721 cells. **(A)** Morphological changes were visualized by fluorescence microscopy after hoechst staining. SMMC-7721 cells were infected with Ad-apoptin or Ad-mock, then stained with Hoechst stain at 12, 24, and 48 h. Nuclear thickening and nuclear fragmentation of Ad-apoptin group increased significantly over time. **(B,C)** SMMC-7721 cells apoptosis were analyzed by flow cytometry after Annexin-V FITC/PI staining. The apoptosis level of SMMC-7721 cells infected with Ad-apoptin was significantly higher than that of other groups; the addition of QVD will cause a significant decrease in the apoptosis level of SMMC-7721 cells infected with Ad-apoptin. **(D)** After the addition of the apoptosis inhibitor QVD to the cells infected with Ad-apoptin, the growth inhibition rate mediated by apoptin was significantly reduced. **(E,F)** Changes in red and green fluorescence, measured by fluorescence microscopy after JC-1 staining. Increased apoptosis results in a decrease in the ratio of red to green fluorescence. Quantitative measurement of changes in the ratio of red to green fluorescence after JC-1 staining. Ad-apoptin clearly altered the mitochondrial membrane potential (MMP), and Ad-apoptin had the strongest ability to induce apoptosis by affecting the MMP. **(G)** Western blotting analysis of caspase-9 and cytochrome C protein expression at different time points on SMMC-7721 cells. The cleaved-caspase-9 and cytochrome C protein level of Ad-apoptin group was higher than Ad-MOCK and control group; after adding QVD, the expression levels of the two proteins were significantly reduced. The scale bar equals 100 μm. Data are presented as mean ± SD (^*^p < 0.05, ^**^p < 0.01, ^***^p < 0.001) when compared with Ad-mock or controls. Data are presented as the mean ± SD (^#^*p* < 0.05, ^##^*p* < 0.01) when compared with Ad-apoptin.

In order to verify whether the apoptin-dependent inhibition of cancer cell growth derived from traditional apoptotic pathways, Hoechst staining of Ad-apoptin-infected cells was further performed (Figure 2A). At 12 h post-infection, apoptin-treating cells started to present apoptotic features, including nuclear fragmentation, while Ad-mock and control groups showed no apoptotic effects. At 24 and 48 h post-infection, Ad-apoptin-infected cells presented a larger amount of nuclear staining, and nuclear fragmentation, when compared to Ad-mock and control groups. The apoptotic features of Ad-apoptin-infected SMMC-7721 cells, including nuclear staining and fragmentation, were significantly reduced after treatment with the apoptosis inhibitor QVD. These data indicate that apoptin may induce apoptosis to inhibit the growth of liver cancer cells.

After qualitative analysis by Hoechst staining, we further analyzed the apoptosis of liver cancer cells, induced by apoptin, by flow cytometry (Figures 2B,C). At 12 h post-infection, the apoptosis rate of cells infected with Ad-apoptin was significantly higher than that of Ad-mock and control groups (*P* < 0.05). This apoptosis rate gradually increased over time, reaching the highest level at 24 h (28.74%). A slight decrease was observed at 48 h post-infection, but degree of this decline was not significant (*P* > 0.05). Again, the apoptin-mediated apoptosis of SMMC-7721 cells was significantly reduced after treatment with QVD. Then MTS analysis was conducted to analyze the effect of apoptosis on SMMC-7721 cells death induced by apoptin (Figure 2D). The cell suppression rate, mediated by apoptin, reached 21.85% at 48 h post-infection after treatmented with QVD. Interestingly, the decreased growth of Ad-apoptin infected cells was overcome significantly upon treatment with QVD. These results reiterate the notion that apoptin mainly inhibits liver cancer cells growth by apoptotic mechanisms.

### Apoptin Induces Apoptosis Through the Intrinsic (Mitochondrial) Pathway

In order to verify whether apoptin inhibits the growth of SMMC-7721 cells by the intrinsic apoptotic pathway, we initially performed JC-1 staining *in vitro* (Figures 2E,F). At 12 h post-infection, apoptin-treating cells showed a decrease in mitochondrial membrane potential. At later time-points, Ad-apoptin-infected cells began to depolarize in large numbers, and the number of apoptotic cells gradually increased over time. JC-1 staining of Ad-apoptin-infected cells gradually changed from initial red aggregates to green monomers. Ad-mock and control cells did not show any significant changes in mitochondrial membrane potential. Upon infection with Ad-apoptin, the mitochondrial membrane potential of SMMC-7721 cells was significantly increased after the simultaneous treatment with QVD, and the ratio of red fluorescence/green fluorescence was higher than that in Ad-apoptin infected cells alone. From the detection of endogenous apoptosis-related protein levels, it was also found that apoptin can significantly increase the expression levels of cytochrome c and cleaved-caspase-9, and the increase becomes more significant with time (Figure 2G). The inhibition of apoptosis can significantly reduce the expression of the two proteins. Hence, apoptin may cause apoptosis in liver cancer cells by activating the intrinsic (mitochondrial) apoptotic pathway.

### Autophagic Activity in the Apoptin-Mediated Growth Inhibtion of Liver Cancer Cells

**Figure 3:**
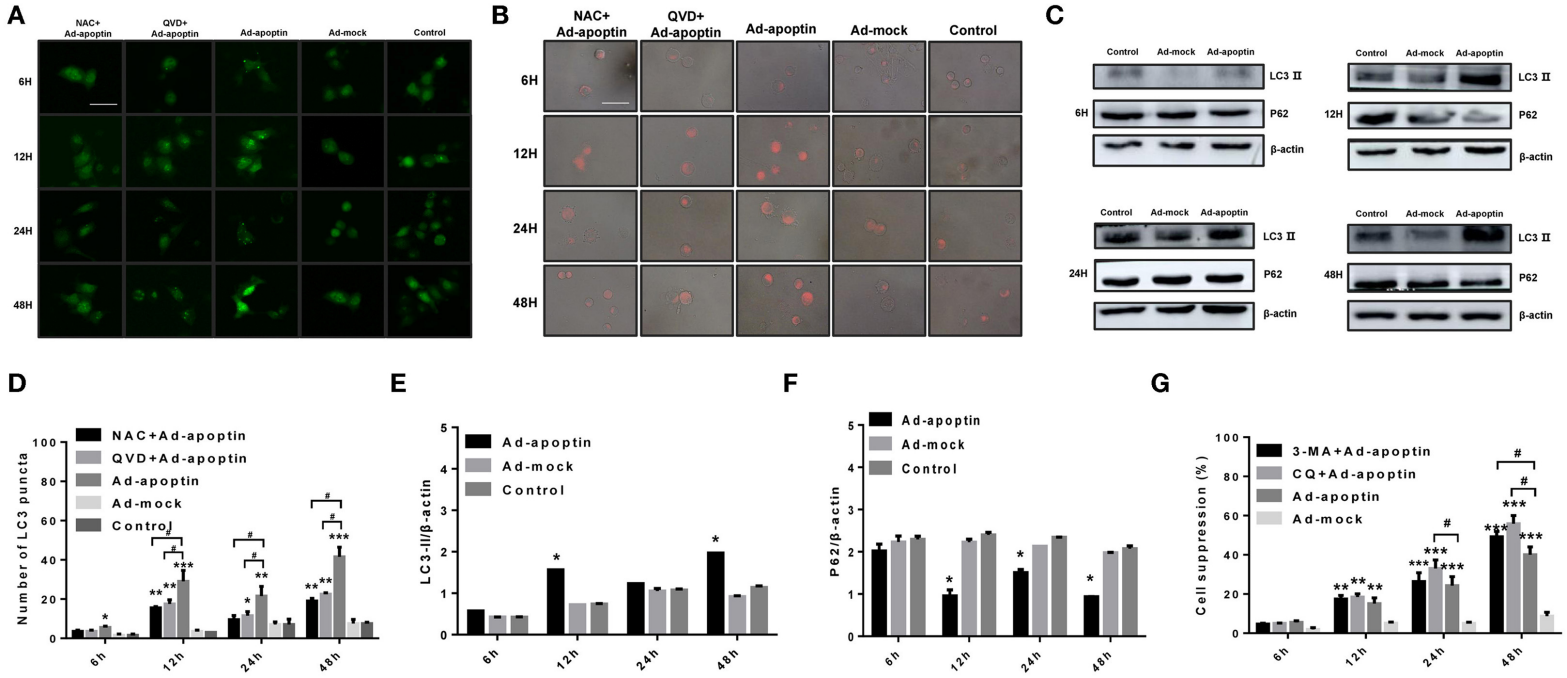
Identification of the effect of apoptin on the changes of autophagy in SMMC-7721 cells. **(A,D)** To evaluate the changes of average number of LC3 puncta in each cell, SMMC-7721 cells were transfected with pEGFP-LC3 and infected with Ad-VP3 and Ad-mock for 6, 12, 24, and 48 h; Apoptin will cause a significant increase in the amount of LC3 puncta, and the addition of QVD and NAC will cause a significant decrease in the amount of LC3 puncta. **(B)** Changes in lysosome counting observed by fluorescence microscopy after LTR staining. SMMC-7721 cells were infected with Ad-apoptin or Ad-mock, then stained with LTR at 6, 12, 24, and 48 h post-infection. The number of lysosomes increased from 12 h; the addition of QVD and NAC will cause a significant decrease in the number of lysosomes. **(C,E,F)** Western blotting analysis of LC3-II and P62 protein expression at different time points on SMMC-7721 cells. The LC3-II and P62 protein level of Ad-apoptin group was higher than Ad-MOCK and control group. **(G)** After addition of the autophagy inhibitor 3-MA and CQ to Ad-apoptin-infected cells, the growth inhibition rate mediated by apoptin was significantly increased. The scale bar equals 50 μm. Data are means ± SD (^*^p < 0.05, ^**^p < 0.01, ^***^p < 0.001) when compared with Ad-mock or controls. Data are shown as the mean ± SD (^#^*p* < 0.05) when compared with Ad-apoptin.

To investigate whether autophagy is altered by apoptin-mediated growth inhibition of liver cancer cells, we initially performed LC3 and LTR staining to evaluate changes in the number of LC3 puncta and lysosome. As shown in Figures 3A,D, the number of LC3 puncta in Ad-apoptin-infected SMMC-7721 cells was relatively low after 6 h of infection. The number of LC3 puncta in Ad-apoptin-infected cells then increased significantly at 12 h post-infection, decreased at 24 h, and reached the highest levels at 48 h. Similar results were obtained in the lysosomal staining experiment (Figure 3B). The Ad-mock and the control groups showed a slow and steady increase in the amount of green and red fluorescence throughout the process. The above results indicate that apoptin affects autophagy while inhibiting the growth of liver cancer cells.

Subsequently, we analyzed the levels of the autophagy-related proteins LC3 and P62 (Figures 3C,E,F). After 12 h of infection with Ad-apoptin, the protein levels of LC3-II in SMMC-7721 cells were significantly higher than those at 6 h (*P* < 0.05). Contrarily, the protein levels of P62 were significantly lower than those of 6 h, and the LC3-II protein levels reduced at 24 h. In contrast, P62 levels were increased, and by 48 h, the LC3-II protein levels reached the highest level throughout the experiment, while P62 reached the lowest point. The levels of LC3-II and P62 proteins in Ad-mock-infected cells were similar to those in the control group. These results again indicate that apoptin affects autophagy while inhibiting the growth of liver cancer cells.

MTS assays were further performed to evaluate whether autophagy has an effect on apoptin-mediated growth inhibition of SMMC-7721 cells. As shown in Figure 3G, the growth rate inhibition induced by apoptin was significantly improved upon treatment with the autophagy inhibitor 3-MA and CQ, and the inhibition rate reached 49.93 and 56.87% at 48 h post-infection. This result indicates that inhibition of autophagy can significantly increase apoptin-induced liver cancer cell death induced.

### Effect of Apoptosis on Autophagy After Apoptin-Induced Liver Cells

In order to verify whether apoptosis induced by apoptin has an effect on autophagy in liver cancer cells, we performed LC3 and LTR staining of Ad-apoptin-infected liver cancer cells (Figures 3A,B). After addition of the apoptosis inhibitor QVD, the number of LC3 puncta and lysosomes in the SMMC-7721 cells from QVD+Ad-apoptin group started to significantly decrease at 12 h. These results suggest that the inhibition of apoptosis in apoptin-treated liver cancer cells can significantly reduce autophagy levels.

**Figure 4:**
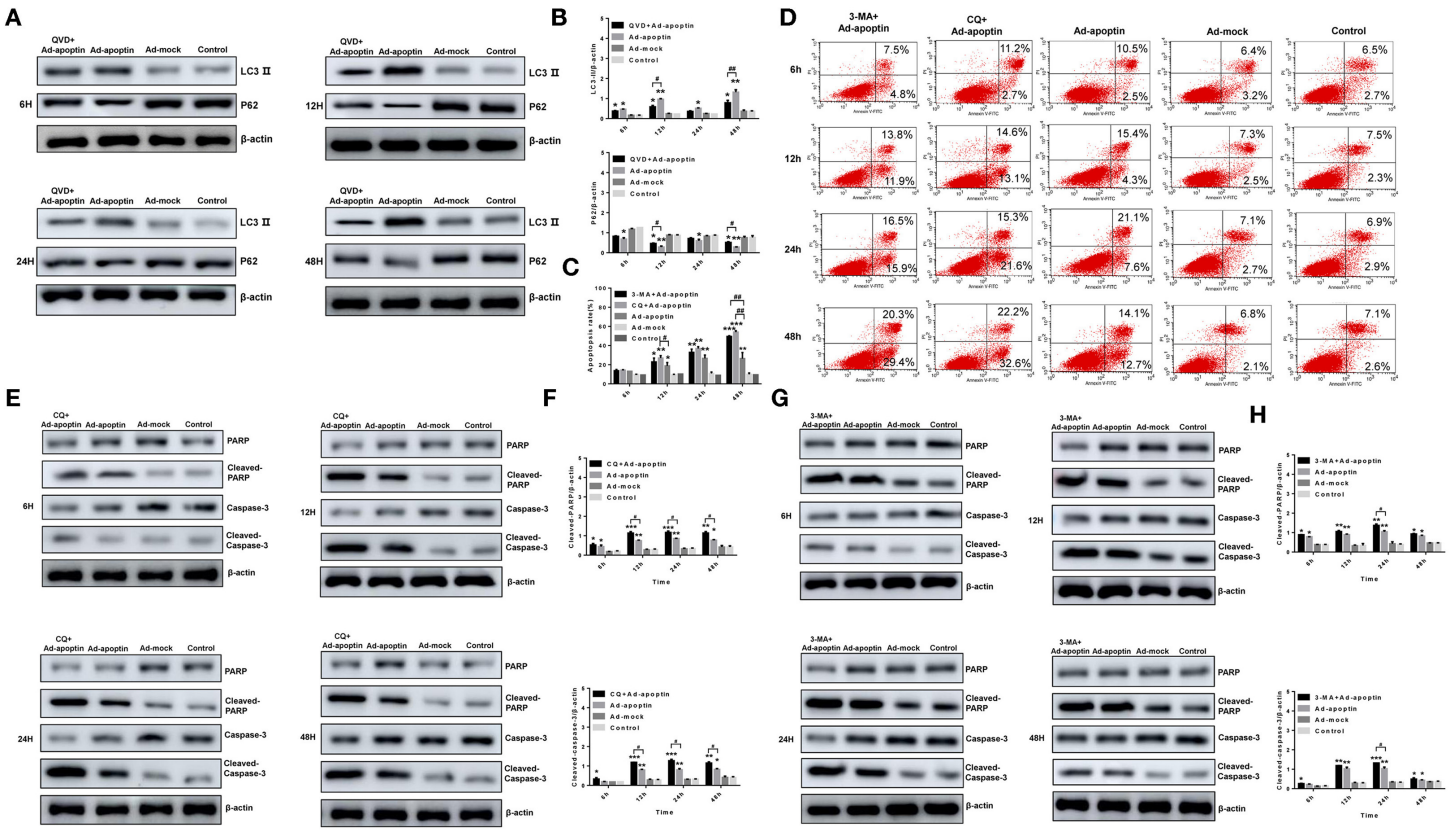
Identification of the relationship between autophagy and apoptosis on apoptin-induced SMMC-7721 cells. **(A,B)** Western blotting analysis of LC3-II and P62 in SMMC-7721 cell extracts. The LC3-IIprotein level of Ad-apoptin group was higher than Ad-MOCK and control group. In contrast, the P62 protein level of Ad-apoptin group was higher than Ad-MOCK and control group. Inhibition of apoptosis in apoptin-treated liver cancer cells can significantly reduce autophagy levels and the difference is most obvious at 12 and 48 h. **(C,D)** Apoptosis analysis by flow cytometry after Annexin-V FITC/PI staining. The apoptosis level of SMMC-7721 cells infected with Ad-apoptin was significantly higher than that of other groups; the addition of 3-MA and CQ will cause a significant increase in the apoptosis level of SMMC-7721 cells infected with Ad-apoptin. **(E,F)** Western blotting analysis of cleaved-PARP and cleaved-Caspase-3 in SMMC-7721 cell extracts. The cleaved-PARP and cleaved-Caspase-3 protein level of Ad-apoptin group was higher than Ad-MOCK and control group; the addition of CQ will cause a significant increase in the expression level of cleaved-PARP and cleaved-Caspase-3 and the difference is most obvious from 12 h. **(G,H)** The addition of 3-MA will cause a significant increase in the expression level of cleaved-PARP and cleaved-Caspase-3 and the difference is most obvious at 24 h. Data are shown as mean ± SD (^*^p < 0.05, ^**^p < 0.01, ^***^p < 0.001) when compared with controls. Data are shown as mean ± SD (^#^*p* < 0.05, ^##^*p* < 0.01) when compared with Ad-apoptin.

The autophagy-related proteins LC3 and P62 protein levels were analyzed after treatment with the apoptosis inhibitor QVD (Figures 4A,B). After infection of SMMC-7721 cells from QVD+Ad-apoptin group, the protein levels of LC3-II were significantly inhibited, and their expression levels were lower than those of the apoptin group. The difference between the QVD+Ad-apoptin group and the apoptin group peaked at 12 and 48 h (*P* < 0.05). The P62 protein levels were significantly higher than that of the apoptin group, and the difference between the QVD+Ad-apoptin group and the apoptin group also peaked at 12 and 48 h (*P* < 0.05). These results show that the inhibition of apoptosis in apoptin-treated cells significantly decreases autophagy levels, and the overall inhibitory effect was more obvious at 12 than 24 h, while the effect at 48 h was the most prominent.

### Effect of Autophagy in Apoptin-Induced Apoptosis

Annexin V-FITC/PI assays were carried out to verify whether autophagy affects the apoptosis induced by apoptin in liver cancer cells. After addition of autophagy inhibitor 3-MA and CQ, the apoptosis level of SMMC-7721 cells in the 3-MA+Apoptin and CQ+Apoptin group increased over time, and it was higher than the apoptosis levels in the Apoptin group alone (Figures 4C,D). The highest levels were reached at 48 h, with apoptotic rates of 48.82 and 54.81%. These rates were significantly higher than those detected in the Apoptin group (*P* < 0.05). These results show that inhibition of autophagy could implement apoptin-mediated apoptosis in liver cancer cells, in a time-dependent manner.

The expression levels of the apoptotic-related proteins PARP and Caspase-3 were also analyzed after treatment with the autophagy inhibitor 3-MA and CQ (Figures 4E–H). At 6 h post-treatment, cleaved-PARP, and cleaved-Caspase-3 levels in SMMC-7721 cells from 3-MA+Apoptin and CQ+Apoptin group were consistently higher than those in the Apoptin group. The difference between the two groups was most significant at 24 h (*P* < 0.05). The above results reiterate the idea that inhibition of autophagy could enhance the apoptosis induced by apoptin in liver cancer cells, with a most obvious effect at 24 h post-treatment.

### Apoptin Induces Mitochondrial Autophagy in Liver Cancer Cells

**Figure 5:**
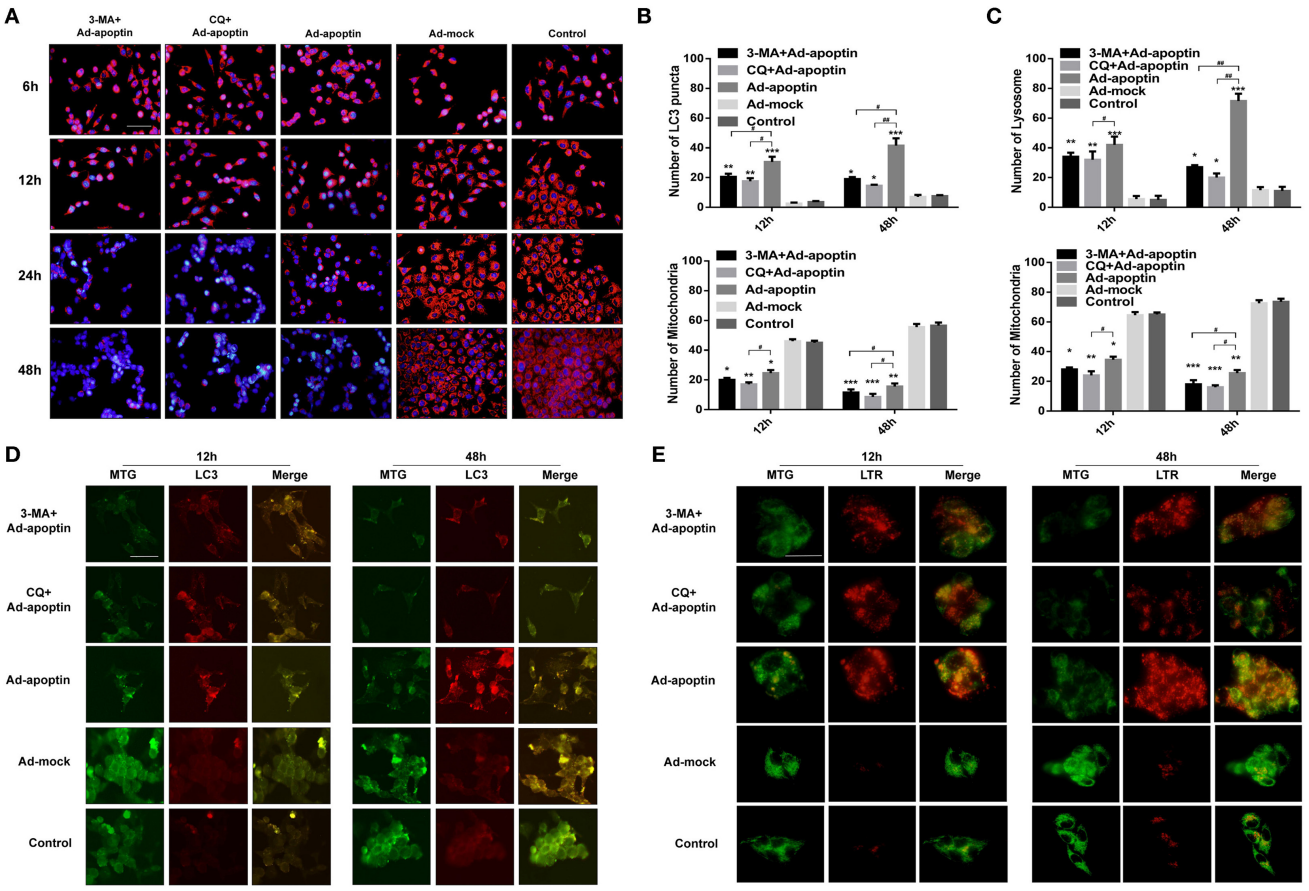
Relationship between autophagy and mitochondria in apoptin-induced SMMC-7721 cells. **(A)** Changes in mitochondrial membrane potential (MMP) observed by fluorescence microscopy, after TMRM and Hoechst staining. SMMC-7721 cells were infected with Ad-apoptin and treated (or not) with the autophagy inhibitor 3-MA and CQ. Cells were further stained with TMRM and Hoechst at 6, 12, 24, and 48 h. Apoptin increases the level of apoptosis of cells and reduces the level of MMP; the addition of 3-MA and CQ will cause a significant increase in the level of apoptosis and a significant decrease in the level of MMP. **(B,D)** MTG and LC3 staining analysis in Ad-apoptin-infected SMMC-7721 cells. Concomitant increase of red fluorescence and the decrease of green fluorescence appears to be caused by apoptin. Upon treatment with 3-MA and CQ, the mitochondrial green fluorescence decreased faster than the LC3 red fluorescence. The bright spots of mitochondria and LC3 are in the same place. At the same time, evaluated the changes of average number of LC3 puncta and mitochondria in each cell. **(C,E)** MTG and LTR staining analysis in Ad-apoptin-infected SMMC-7721 cells. Concomitant increase of red fluorescence and the decrease of green fluorescence appears to be caused by apoptin. Upon treatment with CQ, the mitochondrial green fluorescence decreased faster than the lysosomal red fluorescence. The increase of red fluorescence and the decrease of green fluorescence occurred at same location within the cells. At the same time, evaluated the changes of average number of lysosome and mitochondria in each cell. The scale bar equals 100 μm. Data are shown as mean ± SD (^*^p < 0.05, ^**^p < 0.01, ^***^p < 0.001) when compared with controls. Data are shown as mean ± SD (^#^*p* < 0.05, ^##^*p* < 0.01) when compared with Ad-apoptin.

TMRM and Hoechst staining were further conducted to evaluate whether autophagy has an effect on mitochondrial membrane potential (Figure 5A). At 6 h post-treatment, the mitochondrial membrane potential level of SMMC-7721 cells from the 3-MA+Apoptin and CQ+Ad-apoptin group was lower than that in the Apoptin group and, after 12 h, the 3-MA+Apoptin and CQ+Ad-apoptin group showed a significant decrease in mitochondrial membrane potential. At later times (24 and 48 h), the number of apoptotic cells in the 3-MA+Apoptin and CQ+Ad-apoptin group gradually increased, while the mitochondrial red fluorescence gradually decreased. The difference with apoptin group was most significant at 24 h post-treatment. These results show that inhibition of autophagy may affect the mitochondrial membrane potential of liver cancer cells induced by apoptosis.

MTG and LC3/LTR staining were carried out to further understand the relationship between autophagy and mitochondria homeostasis (Figures 5B–E). In these two staining experiments, we selected 12 and 48 h with higher autophagy levels for the experiment. At 12 h, the mitochondrial green fluorescence of cells in the Ad-apoptin group decreased compared with the control group, while the LC3 and lysosomal red fluorescence increased. At 48 h post-infection, the green fluorescence decreased more significantly, while the red fluorescence also increased more significantly. The cells in the Ad-mock group behaved similar to the control group throughout the process, with no significant difference. After adding 3-MA or CQ, we observed that the green and red fluorescence was significantly weakened at 12 and 48 h, which was even weaker than those in the Apoptin group. Interestingly, the increase of red fluorescence and the decrease of green fluorescence caused by apoptin occurred at same location within the cells. After addition of 3-MA or CQ, the mitochondrial green fluorescence decreased faster than the LC3 and lysosomal red fluorescence. These result indicate that apoptin may induce mitochondrial autophagy while inducing apoptosis in liver cancer cells.

### ROS Affects the Inhibitory Effect of Apoptin on Liver Cancer Cells

**Figure 6:**
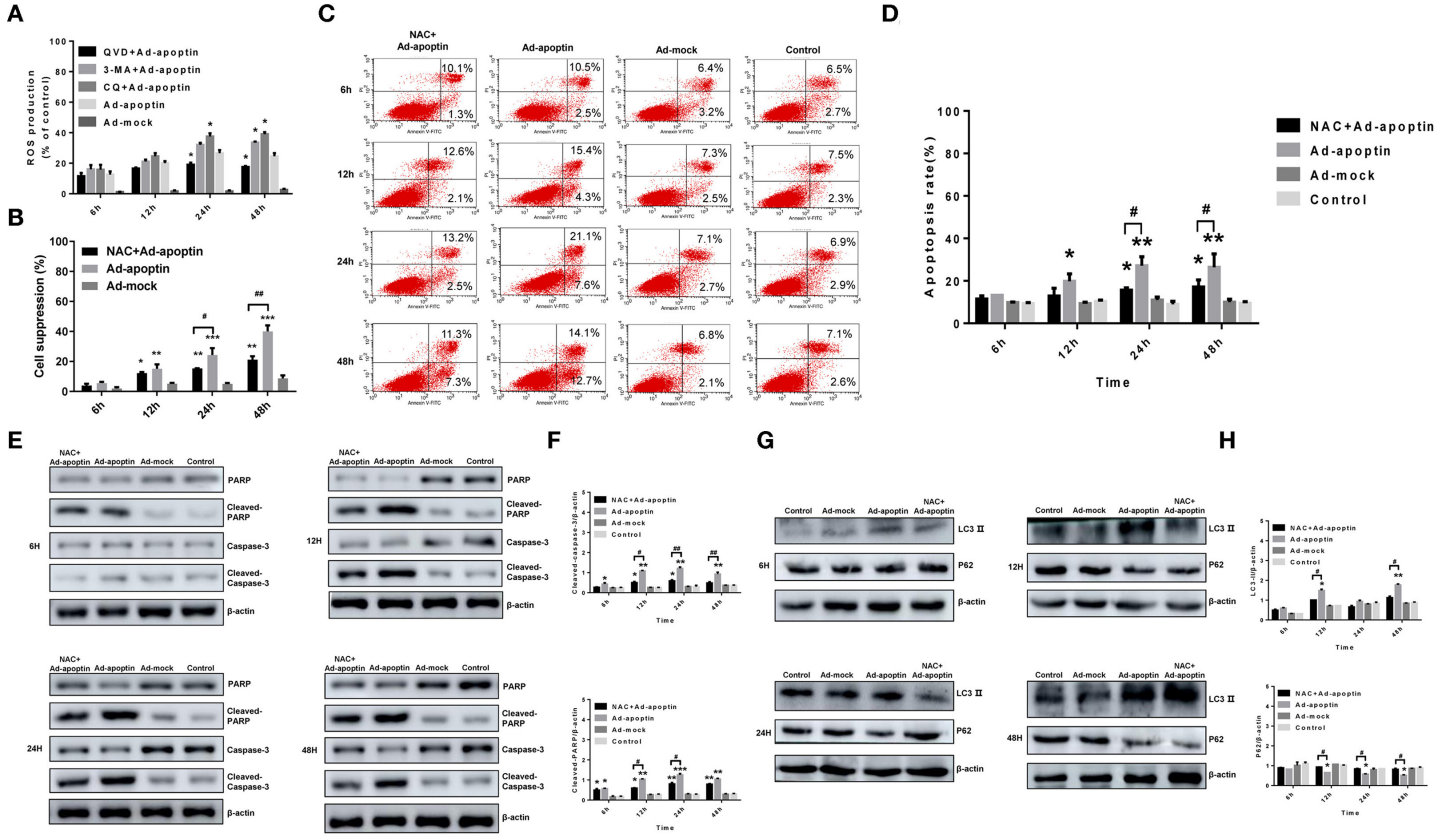
Effects of ROS toward apoptosis and autophagy in apoptin-induced SMMC-7721 cells. **(A)** ROS levels in SMMC-7721 cells were analyzed by flow cytometry after DHR staining. Upon treatment with QVD, ROS levels in the QVD+apoptin group decreased to varying degrees. Upon addition of autophagy inhibitor 3-MA and CQ, ROS levels in the CQ+Ad-apoptin group was significantly higher than that in the untreated apoptin group starting from 24 h post-infection; and ROS levels in the 3-MA+Ad-apoptin group was significantly higher than that in the untreated apoptin group starting at 48 h post-infection. **(B)** After addition of the ROS inhibitor NAC to Ad-apoptin-infected cells, the growth inhibition rate mediated by apoptin was significantly decreased. **(C,D)** SMMC-7721 cells apoptosis were analyzed by flow cytometry after Annexin-V FITC/PI staining. The apoptosis level of SMMC-7721 cells infected with Ad-apoptin was significantly higher than that of other groups; the addition of NAC will cause a significant decrease in the apoptosis level of SMMC-7721 cells infected with Ad-apoptin. **(E,F)** Western blotting analysis of cleaved-PARP and cleaved-Caspase-3 in SMMC-7721 cell extracts. Inhibition of ROS can decrease apoptin-induced apoptosis in liver cancer cells and then increases over time. **(G,H)** Western blotting analysis of LC3-II and P62 in SMMC-7721 cell extracts. Inhibition of ROS in apoptin-treated liver cancer cells can significantly reduce autophagy levels. Data are shown as mean ± SD (^*^p < 0.05, ^**^p < 0.01, ^***^p < 0.001) when compared with Ad-mock or controls. Data are shown as mean ± SD (^#^*p* < 0.05, ^##^*p* < 0.01) when compared with Ad-apoptin.

To investigate whether apoptin inhibits the liver cancer cells growth and affects ROS levels, we conducted flow cytometry analyses based on DHR staining (Figure 6A). Upon infection with Ad-apoptin, ROS levels significantly increased *in vitro*. At 6 h post-infection, ROS levels in the Apoptin group were significantly higher than those in the Ad-mock group (*P* < 0.05). Over time, ROS levels gradually increased, reaching a peak at 24 h post-infection. ROS levels in the Ad-mock group were similar to those in the control group (*P* > 0.05). Upon treatment with QVD, the ROS levels in the QVD+Ad-apoptin group decreased to varying degrees. After adding 3-MA and CQ, the ROS levels in the CQ+Ad-apoptin group were significantly higher than those in the Apoptin group, starting at 24 h post-infection (*P* < 0.05); and the ROS levels in the 3-MA+Ad-apoptin group were significantly higher than those in the Apoptin group at 48 h post-infection. These results indicate that apoptin significantly affects ROS levels in liver cancer cells, and this effect is closely related to apoptosis and autophagy.

MTS assays were conducted to further examine whether ROS may affect apoptin-mediated growth inhibition in liver cancer cells (Figure 6B). Upon treatment with the ROS inhibitor NAC, the inhibitory rate of apoptin was significantly reduced in Ad-apoptin infected cells. This result resembles those obtained after inhibition of apoptosis. This indicates that inhibition of ROS may significantly reduce the apoptin-mediated growth inhibition of liver cancer cells.

### ROS Affects Apoptin-Induced Apoptosis and Autophagy on Liver Cancer Cells

To verify whether ROS affects apoptosis, the apoptin-induced apoptosis of liver cancer cells was quantitatively analyzed using ROS inhibitor NAC. After infection with Ad-apoptin, apoptosis in SMMC-7721 cells was significantly reduced after the addition of the ROS inhibitor NAC. At 6 h post-treatment, apoptosis started to be lower than the untreated Apoptin group. At 12 h, it was significantly lower than the apoptin group, reaching 18.63% (Figures 6C,D) at 48 h after NAC treatment. These observations indicate that ROS inhibition can effectively reduce the apoptin-mediated apoptosis in liver cancer cells.

The levels of apoptosis-related proteins PARP and Caspase-3 were then analyzed in NAC-treated liver cancer cells (Figures 6E,F). A significant decrease on the levels of cleaved-PARP and cleaved-Caspase-3 was observed in the Apoptin group after treatment with NAC. Compared with the untreated group, this reduction on protein levels was more significant at 12, 24, and 48 h after NAC treatment (*P* < 0.05). The results reiterate that ROS inhibition can effectively reduce the apoptosis, mediated by apoptin, in liver cancer cells.

Subsequently, LC3 and LTR staining of apoptin-treating cells was carried out to verify whether apoptin-mediated ROS production had an effect on autophagy (Figures 3A,B). After 6 h of treatment with the ROS inhibitor NAC, the number of LC3 puncta and lysosomes in treated apoptin group was smaller than that of the untreated group. The amount of red fluorescence was also significantly less than that of apoptin group at 12 h of NAC treatment. These results suggest that ROS inhibition in apoptin-activated liver cancer cells significantly reduced the level of autophagy *in vitro*.

The levels of autophagy-related proteins LC3 and P62 were also examined after NAC treatment (Figures 6G,H). The protein levels of LC3-II were significantly inhibited in the NAC+Ad-apoptin group, and their expression levels were lower than those in the control group. The difference between the QVD+Ad-apoptin group and the apoptin group was the highest at 12 and 48 h treatment (*P* < 0.05). In contrast, the P62 protein levels were significantly higher than that of the control group and, coincidently, the difference between the NAC+Ad-apoptin group and the apoptin group was the highest at 12 and 48 h treatment (*P* < 0.05). This suggests that ROS inhibition in apoptin-treated cells significantly decreases autophagy and the overall inhibitory effect was more obvious at 12 h than 24 h treatment, with a peak effect at 48 h.

## Discussion

Cancer is a major malady that threatens human health. In recent years, the number of patients with various tumors has gradually increased, and the mortality rate has also increased. In China, the incidence of liver cancer in men is only second to lung cancer, and the incidence of liver cancer in women is only second to breast cancer (1). The death rate due to liver cancer is also only second to the above two cancers (1).

The adenovirus genome is small and easy to modify. After inserting a foreign gene into an adenovirus vector, it can express a large amount of ectopic protein very efficiently (29). Therefore, adenovirus has great potential in the field of tumor therapy. In this study, a recombinant adenovirus Ad-apoptin was constructed using a human type 5 adenoviral vector expressing apoptin. This delivery system has been shown to efficiently produce apoptin protein *in vitro* (27).

Apoptin is a protein that specifically induces apoptosis in tumor cells. Since this protein is not mediated by p53 or inhibited by Bcl-2 overexpression, it has been considered as a novel anti-tumor biological agent (10–12). However, the autophagy-mediated activation of apoptin and its interaction with apoptosis have not been precisely elucidated. In this study, human liver cancer cells were infected with recombinant adenoviruses expressing human apoptin (Ad-apoptin) and, by detecting whether apoptin can induce apoptosis and/or autophagy in liver cancer cells, a putative correlation between these cell death mechanisms was investigated.

Firstly, the inhibitory effect of apoptin on human liver cells was analyzed by crystal violet staining and MTS assay. The results showed that Ad-apoptin had a relative killing effect in liver cancer cells. Ad-apoptin was also able to inhibit tumor cells over time, reaching a peak ~40% at 48 h post-infection. So, apoptin can effectively inhibit the growth of human liver cancer cells, in a time- and dose-dependent manner. In other cancer cell models including SW1116 (colon) (30), A549 (lung) (31), MCF-7 (breast) (32), PC-3 (prostate) (33), SGC7901 (gastric) (34), and A375 (melanoma) (27), Ad-apoptin had a significant inhibitory effect (growth rate inhibition of ~30%). In our current study, the inhibition rate in human liver cancer cells were up to ~40%, suggesting that apoptin may be more sensitive to liver cancer cells than to other tumor cells and, therefore, apoptin has a greater potential to become a therapeutic lead for liver cancer cells. Based on tumor inhibition assays *in vivo*, we have also discovered that apoptin shows a very significant inhibitory effect in liver cancer cells. Interestingly, apoptin overexpression was not only capable of inhibiting tumor growth, but also promoted (and improved) animal survival. Hence, apoptin is not only able to inhibit the growth of liver cancer cells *in vitro*, but also has a significant antitumor effect *in vivo*.

After Hoechst staining and Annexin V-FITC/PI flow analysis, we found out that the apoptosis level of Ad-apoptin-infected liver cancer cells started to increase at 12 h post-infection. Over time, the apoptosis level of Ad-apoptin group gradually increased, and peaked at 24 h post-infection, reaching ~30%, but decreased slightly at 48 h (degree of decline was not significant; *P* > 0.05). Upon addition of the apoptosis inhibitor QVD, the apoptosis of liver cancer cells was significantly reduced. This result is similar to other previous studies, in which apoptin is able to kill a variety of tumor cells mainly by apoptotic mechanisms (35).

Changes on mitochondrial membrane potential were subsequently analyzed by JC-1 and TMRM staining as means to understand whether apoptin can inhibit the growth of liver cancer cells by the intrinsic apoptotic pathway. Starting at 12 h post-infection, mitochondria began to damage after cells are infected with Ad-apoptin. At later times, Ad-apoptin infected cells displayed a depolarization phenomenon and, upon increasing the time of infection, apoptotic cells increased gradually. Consistently, TMRM staining displayed similar results. These observations indicate that apoptin can inhibit cell growth by altering MMP, which might lead to apoptosis of liver cancer cells by the intrinsic apoptotic pathway. We also detected mitochondrial apoptosis-related proteins, caspase-9, and cytochrome c in this study, and found that changes in the two proteins are directly proportional to changes in the level of apoptosis; at the same time, the addition of QVD could significantly inhibit the two proteins expression. Studies have shown that apoptin expression in human osteosarcoma cells (Saos-2) can lead to caspase activation (36). Using FADD and caspase-8 deficient Jurkat T cells, other reports have suggested that apoptin-mediated cell death is independent of death receptors (19). These observations corroborate the results of our current study.

It has been increasingly accepted that the occurrence and development of tumors are not only due to uncontrolled cell proliferation and abnormal differentiation, but also related to the imbalance of tumor cell apoptosis (37–39). However, apoptosis is far from being the only mechanism that determine cell fate. In recent years, autophagy has been shown to co-regulate cell death (40). In some cases, autophagy may inhibit apoptosis or, contrarily, autophagy itself can induces cell death or even interact with apoptosis to induce cell death as a “backup” mechanism (41).

Here, LC3 and LTR staining and analysis of autophagy key proteins (LC3 and P62) revealed that apoptin inhibits cell growth and affects autophagy. In order to analyze whether autophagy affects apoptin-mediated inhibition of liver cancer cells, an MTS assay was performed. The results showed that the inhibitory rate of apoptin was significantly increased after adding autophagy inhibitor 3-MA and CQ to cells infected with ad-apoptin. The inhibition rate reached 49.93 and 56.87% at 48 h, which were significantly higher than that of Ad-apoptin alone. This result indicates that inhibition of autophagy can significantly increase apoptin-induced liver cancer cells death.

Studies have shown that cellular stress can cause autophagy, which can also lead to apoptosis (42). The various roles of these two mechanisms can be defined as the following: (i) autophagy can be used as a survival mechanism by limiting stress caused by environmental factors, and maintaining normal cell function to antagonize apoptosis (43); (ii) autophagy can also act as a promoter of apoptosis by maintaining ATP levels required for ATP-dependent apoptosis (44); (iii) autophagy can synergize apoptosis by entering into the apoptotic process and modulating caspase activity (45–47); and (iv) autophagy can also act as a “backup mechanism,” involved in the transport of damaged organelles, such as mitochondria (i.e., mitochondrial autophagy) (48–50).

Here we also conducted a preliminary study correlating apoptin-induced autophagy and apoptosis in liver cancer cells. We have discovered that apoptosis inhibition can significantly reduce autophagy levels in apoptin-induced liver cancer cells. Similarly, autophagy inhibition can enhance apoptin-induced apoptosis of liver cancer cells, in a time-dependent manner. In addition, the effect of autophagy in MMP was also analyzed, and it was verified that autophagy inhibition may have a certain effect on apoptin-induced changes in MMP. In this case, the decrease of MMP was more prominent over time. Next, we analyzed the relationship between autophagy and mitochondrial activity/homeostasis by organelle staining (i.e., mitochondria and lysosomes). We initially observed a co-localization of mitochondria and LC3/lysosomes. Upon addition of 3-MA or CQ as an autophagy inhibitor, the reduction rate of green fluorescence in mitochondria was higher than that of red fluorescence in lysosomes. Hence, apoptin may induce apoptosis and mitochondrial autophagy in liver cancer cells. Altogether, autophagy appears to play a protective role over time, while when the apoptosis level is at the highest at 24 h, the apoptosis effect may exceed the threshold of the protective role played by autophagy and occupy a dominant position, so the autophagy is at the lowest level.

ROS is mainly formed in the mitochondria, and it plays an important role in mitochondrial activity (51). In fact, mitochondrial defects can significantly increase the level of ROS.

In this study, we found that apoptin-induced apoptosis and autophagy *in vitro* also promoted an effect in the mitochondria. Therefore, we analyzed whether apoptin-mediated inhibition of ROS production and cancer cell growth occurs concomitantly. After DHR staining, the level of ROS in apoptin-treating cells was significantly increased, in a time-dependent manner. After the addition of the apoptosis inhibitor QVD, the level of cellular ROS abruptly decreased. These levels were significantly lower than that of the untreated apoptin group at 12 h post-infection, and the difference was more significant at 24 and 48 h (*P* < 0.05). After adding autophagy inhibitor 3-MA and CQ, the levels of cellular ROS were significantly higher than that of the untreated apoptin group at 24 h post-infection (treated with CQ), and the difference was more significant at 48 h (treated with 3-MA and CQ) (*P* < 0.05). It is also shown that apoptin can significantly modulate ROS in liver cancer cells, and this is closely related to apoptosis and autophagy, since the higher the level of ROS, the greater the influence of autophagy and apoptosis In fact, after treatment with ROS inhibitor NAC, apoptin-induced apoptosis, and autophagy in liver cancer cells were significantly decreased.

## Conclusions

In summary, apoptin can significantly inhibit the growth of human liver cancer cells, mainly causing the death of liver cancer cells in an apoptotic manner, but with a significant impact on autophagy. Autophagy plays a protective role but, upon apoptosis activation, the apoptotic effect may exceed the threshold of protective effect of autophagy. Still, both autophagy and apoptosis in liver cancer cells involve an increase in mitochondrial ROS levels, resulting in loss of mitochondrial transmembrane potential. Therefore, our study suggests that ROS can be a key factor in linking apoptin-induced autophagy and apoptosis in liver cancer cells.

## Data Availability Statement

The data that support the findings of this study are available from the corresponding author upon reasonable request.

## Ethics Statement

The animal study was reviewed and approved by the Institutional Animal Care and Use Committee (IACUC) of the Chinese Academy of Military Medical Science (Changchun, China) (10ZDGG007).

## Author Contributions

YL, XLi, LS, and NJ: conceived and designed the experiments. YL, YZ, JF, WL, SL, XLiu, ZL, GS, and NJ: performed the experiments. YL, XLi, LS, and NJ: analyzed the data. YZ, CS, SL, JC, and BB: contributed reagents, materials, and analysis tools. YL and XLi: wrote the paper. All authors: read and approved the final manuscript.

## Conflict of Interest

The authors declare that the research was conducted in the absence of any commercial or financial relationships that could be construed as a potential conflict of interest.
